# Non-Equilibrium Hyperbolic Transport in Transcriptional Regulation

**DOI:** 10.1371/journal.pone.0021558

**Published:** 2011-07-06

**Authors:** Enrique Hernández-Lemus, María D. Correa-Rodríguez

**Affiliations:** 1 Computational Genomics Department, National Institute of Genomic Medicine, México, México; 2 Center for Complexity Sciences, National Autonomous University of México (UNAM), México, México; 3 Physics Department, School of Sciences, National Autonomous University of México (UNAM), México, México; Universita' del Piemonte Orientale, Italy

## Abstract

In this work we studied memory and irreversible transport phenomena in a non-equilibrium thermodynamical model for genomic transcriptional regulation. Transcriptional regulation possess an extremely complex phenomenology, and it is, of course, of foremost importance in organismal cell development and in the pathogenesis of complex diseases. A better understanding of the way in which these processes occur is mandatory to optimize the construction of gene regulatory networks, but also to connect these networks with multi-scale phenomena (e.g. metabolism, signalling pathways, etc.) under an integrative Systems Biology-like vision. In this paper we analyzed three simple mechanisms of genetic stimulation: an instant pulse, a periodic biochemical signal and a saturation process with sigmoidal kinetics and from these we derived the system's thermodynamical response, in the form of, for example, anomalous transcriptional bursts.

## Introduction

### Transcriptional kinetics, memory functions and hyperbolic differential equations

Messenger RNA transcription from a DNA template is a chemical process regulated by different genes and their products. Being this the case, a variety of physicochemical interactions abound between genetic transcripts abundance and it is a recognized fact that such complex processes are behind the ultimate mechanisms of cell function. Genome-wide transcriptional Expression Analysis (GEA) has allowed us to go well beyond studying gene expression at the level of individual components of a given process by providing global information about functional connections between genes, mRNAs and the related regulatory proteins. GEAs have greatly increased our understanding of the interplay between different events in gene regulation and have pointed out to previously unappreciated biological functional relations, such as the coupling between nuclear and cytoplasmic transcription and metabolic processes [1]. GEA also revealed extensive communication within regulatory units, for example in the organization of transcription factors into regulatory motifs. However, these coupling phenomena are usually studied by means of probabilistic modeling. Even if such stochastic models have been extremely useful, there is a lack for a phenomenological explanation and the corresponding theoretical framework. A first step towards this goal would be achieved by understanding the thermodynamical basis of such sets of coupled biochemical reactions [2].

In the case of transcriptional regulation inside the cell, the thermodynamic analysis faces various challenges, mainly related with the cell being a *small* system (hence the role of fluctuations and irreversible couplings gain a great deal of importance), and with the non-linear, non-local nature of chemical reactions. An appealing scenario to consider is the cellular behavior of the RNA-polymerase molecule (RNApol). RNApol is an enzyme that moves along the DNA to produce a newly synthesized mRNA molecule. It has been mentioned that RNApol extracts energy from its surrounding thermal bath (i.e. the cellular environment) to move, and at the same time uses bond hydrolysis to insure that only thermal fluctuations that lead to *forward* movement are captured. RNApol then serves as an out-of-equilibrium thermal rectifier. The complex dynamics behind even this (relatively) simple model of transcription demonstrate the necessity for a non-equilibrium thermodynamical characterization that includes the possibility to deal with fluctuations in small systems. Systems outside the realms of the thermodynamic limit are characterized by large fluctuations and hence stochastic effects are to be considered. An extremely important question in contemporary thermal physics lies in the connections between probability and thermodynamics. In fact, a developed theory exists, called *mesoscopic nonequilibrium thermodynamics* (MNET) [3] which specifically addresses the issue by considering the stochastic nature of the time evolution of small non-equilibrium systems, in a context which is extremely close to our work. MNET for small systems could be understood as an extension of the equilibrium thermodynamics of small systems developed by Hill and co-workers [4]–[6].

The way in which stochasticity is taken into account is by means of recognizing that scaling down the description of a physical system brings up energy contributions that are usually neglected in thermodynamical descriptions. Recall that any reduction of the spatio-temporal scale description of a system would entail an increase in the number of non-coarse grained degrees of freedom. These degrees of freedom could be related with the extended variables in Extended Irreversible Thermodynamics [7], but they could also be more microscopic in nature, such as colloidal-particle velocities, orientational states on a quasi-crystal, and so on. Hence, in order to characterize such variables, MNET considers that there exist a set 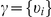 of such non-equilibrated degrees of freedom. 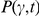 is the probability that the system is at a state given by 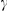 at time 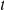. If one assumes [8], [9], that the evolution of the degrees of freedom could be described as a diffusion process in 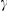-space, then the corresponding Gibbs equation could be written as:
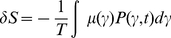
(1)

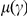
 is a generalized chemical potential related to the probability density, whose time-dependent expression could be explicitly written as:
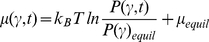
(2)or in terms of a *nonequilibrium work term* 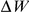:
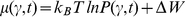
(3)


The time-evolution of the system could be described as a generalized diffusion process over a potential landscape in the space of mesoscopic variables 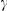. This process is driven by a generalized mesoscopic-thermodynamic force 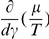 whose explicit stochastic origin could be tracked back by means of a Fokker-Planck-like analysis [3], [10]. One important setting where MNET seems appropriate is the case of activated processes, like a system crossing a potential barrier. Chemical reactions (and biochemical reactions like the ones involved in gene regulation too!) are clearly in this case. According to [8] the diffusion current in this 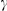-space could be written in terms of a local fugacity defined as:
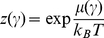
(4)and the expression for the associated flux will be:
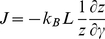
(5)


L is an Onsager-like coefficient. After defining a *diffusion coefficient* 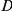 and the associated affinity 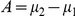, the integrated rate is given as:
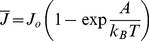
(6)with 
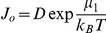
.

One is then able to see that MNET gives rise to nonlinear kinetic laws like Eq. 6. MNET then provides a systematic and straightforward way to obtain stochastic non-equilibrium dynamics (Fokker-Planck equations) starting from the equilibrium properties of the system. Its applications include nonlinear transport phenomena and activation processes [3] that as we will see later, are the cornerstones of the thermodynamic characterization of transcriptional regulation presented here. In this context MNET has been applied successfully in the past in biomolecular processes at (or under) the cellular level of description [3].

In that scenario, non-linear kinetics have been used to express, for example RNA unfolding rates as *diffusion currents*, modeled via transition state theory, giving rise to Arrhenius-type non-linear equations [11]. In that case the current was proportional to the chemical potential difference, so the entropy production was quadratic in that chemical potential gradient. We will re-examine these kind of dependency later when discussing gene expression kinetics. In brief, the MNET approach is based in the generalization of the definition of chemical potential to account for additional *mesoscopic* variables and the assumption that the dynamic evolution of these added degrees of freedom could be described by means of a diffusion process, in order to formulate the corresponding Gibbs equation. By doing so we notice that the time evolution of nonequilibrium systems mimics a generalized diffusion process over a potential landscape in the space of mesoscopic variables [3]. Later we will present a *Black Box Model* of transcriptional regulation that is inspired in these same lines of thought as MNET. This is so since we will be studying transcriptional regulation as a generalized transport process in a mesoscopic scale driven by activation kinetics. Transport at a mesoscopic scale is affected by forces of different nature that characterized it to be intrinsically non-linear and influenced by fluctuations. We will show later that a means to analyze transport under such conditions lies in the consideration of memory processes.

In the other hand, regulatory network analyses have indicated that different levels of gene expression are strongly coupled. An important setting in which cooperativity appears is the phenomenon of anomalous transcriptional bursts (ATBs) that could be observed by noticing that protein production often occurs in bursts, each due to a *single* promoter or transcription factor binding event. Although mRNA concentrations can be modified by altering synthesis and/or degradation rates, the dynamics of the transition to a new concentration are highly dependent on the regulatory mechanisms related to mRNA stability. There are a number of different scenarios or transcriptional strategies following environmental change or differentiation cycles, these in turn reflect different degrees of compromise between speed of response and cost of synthesis [12]. It comes as no surprise that non-local irreversible processes naturally arise within such complex biochemical settings [13], [14]. It has also been possible also to deal with complex chemical kinetics by means of a probabilistic thermodynamics approach [8] if one consider chemical reactions as generalized diffusion processes along internal coordinates. Reactions are thus viewed as diffusion processes through a potential barrier whose minima are related with the initial and final states of the reactions (i.e. reactants and products) in which a *particle* of the *activated complex* [15] crosses the potential barrier between those two states. This allows to write the diffusive current (the flux) as a linear law that relates the local reaction rate and the gradient of the chemical potential which is the thermodynamic driving force in the state of internal variables [8]. This approach is very close to the one that we will follow later (e.g. in equation 13) when we consider transcriptional fluxes as generalized transport processes to be modeled as linear laws with a memory kernel. The fluctuating hydrodynamics approach in reference [8] will also reveal to be useful to connect probability (as given by generalized Langevin dynamics) with nonequilibrium thermodynamics and in particular with the notion of a generalized entropy as the thermodynamic potential [3], [7], [16]. As we know, classical theories for memory effects have been successful in connecting transport processes with fluctuations and probability. A well known general account is given in reference [7], here the authors show precisely the equivalence of theories for transport with memory with generalized entropies such as the one used in the present paper and in most of the extended irreversible thermodynamics formulations. A more recent approach to the connection between classic Langevin dynamics and non-linear thermodynamics was formulated by Qian [16] in the context of MNET for *single macromolecule* description. There are also several other important examples, which contributions range from fluctuating hydrodynamic models [17], information theoretical approaches [18], memory function formalisms [19] and even projection operator techniques [20].

Feedback between mRNA and protein production may result in kinetic bistability and oscillations. Bistability in gene transcription is believed to be widely used as a key ingredient in the regulation of cellular activity. The physiological role of kinetic oscillations in gene transcription is still an open question [14], [21]. However, the presence of non-linear transport processes in these reactive systems could be represented by means of hyperbolic-type differential equations (HDE). Within the context of transport theory (especially linear response theory) it is possible to analyze the connection between HDEs and so-called *Memory functions*. Memory functions are thus useful to unify coherent and incoherent transport. These limiting cases arise as a consequence of two extreme situations in the dynamic evolution of systems: oscillations and decays. In fact, one of the most interesting questions in physicochemical dynamics is the relation between microscopic short-time oscillations and macroscopic long-time decays. This relation is deeply connected with questions such as non-locality and irreversibility, facts that are behind the hyperbolic structure in transport differential equations [22], [23]. Memory functions are mathematical constructs that enable that wave-like and exponential solutions coexist. This could be observed if we consider that the *wave equation* 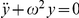 which gives rise to periodic oscillations, and the *damping equation* 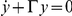 which causes exponential decays; could be unified by means of the so-called memory equation:
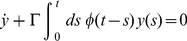
(7)


Here single-dotted quantities are first time derivatives, double-dotted quantities second time derivatives, 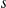 is a parameter and 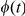 is called the *memory function* whose role is connecting 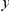 at all times-past to its present derivative 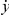. It is noticeable that if 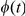 is a delta function or a constant (equal to 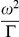) equation 7 reduces to either the wave equation or the damping equation, respectively [22]. However if the memory function is neither a delta function nor a constant, but it is defined as: 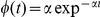 with 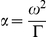, the time evolution of 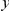 is a wave at short times and a decay at larger times and is, in this sense an interesting unification between these two disparate behaviors. Notice that if we use 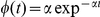 in equation 7 and apply time derivative, we obtain the equation of motion for the damped harmonic oscillator. It is also important to stress that memory functions are a means of unifying coherent (wave-like) and incoherent (or diffusive) transport processes. Real kinetic processes (like the ones present in reaction-diffusion fronts) often present this combination of wave-like propagation with diffusive evolution, as has been known for decades [24].

## Analysis

### Thermodynamic formalism

It is customary in non-equilibrium thermodynamics to assume that a generalized entropy-like function 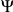 exists, which may be written in the form [7], [25]:
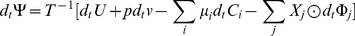
(8)


Eq. 8 is a formal extension of the Gibbs relation of equilibrium thermodynamics.The quantities appearing therein are as usual: 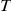 is the local temperature, 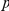 and 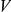 the pressure and volume, etc. 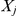 and 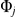 are extended thermodynamical fluxes and forces [7]. For a multicomponent mRNA mixture (under fixed volume and pressure), the set of relevant variables consists in the temperature 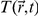 and concentration of each gene species 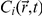 as the slow varying (classical) parameters set and the *mass flux* of these species 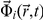 as fast variables. These latter variables will take into account the presence of inhomogeneous regions (concentration domains formed because of the gene regulatory interactions) to correct the predictions based on the local equilibrium hypothesis. The non-equilibrium Gibbs free energy for a mixture of 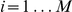, mRNA transcripts reads [2]:
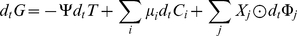
(9)


Gene expression is of course a chemical process. In principle, then, it must be useful to consider the extent of reaction 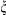, hence 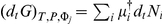 is rewritten by using the *stoichiometric coefficients* 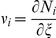 and the *chemical affinities* 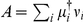. The stoichiometric coefficients and the chemical affinities could be defined likewise for a set of (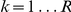) regulatory interactions (considered as *chemical reactions*) as follows:
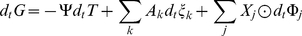
(10)or
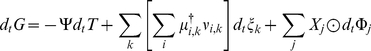
(11)


#### Biochemical kinetics in Gene Regulation: A Black-Box model

In most cases, the explicit stoichiometry of the regulatory interactions is unknown and in the vast majority of the already studied cases the reactions are given on a one-to-one basis, i.e. one molecule of a transcription factor on each gene-transcription site (or one molecule of each kind of transcription factor in the case of multi-regulated gene targets). Given this, we will assume 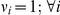. In this so-called *diluted* case we have that the extent of each reaction is then proportional to the concentration rate of change and we recover the non-reactive regime similar to that given by Eq. 9. It is important to stress that this approximation is not a disparate one, given the fact that usual DNA/RNA concentrations within the cells are in the picomolar-nanomolar regime. Also, as an example, of the almost 30,000 different genes in humans just a small number of these (about 1000–1500) are known to be transcription factors. Nevertheless in order to take into account the scarce yet important gene regulatory interactions (albeit in an indirect *Black-Box* manner) we retain the generalized force-flux terms to get:
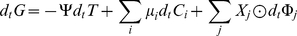
(12)


Since gene regulation occurs within the cell, it is possible to relate an internal *work* term with the regulation process itself, being this a *far from equilibrium* contribution. This contribution is given by the generalized force-flux term (third term in the r.h.s. of Eq. 12). This is so as gene regulation often does not occur *in situ* and also since is a means to take into account the changes in the local chemical potentials that cause the long tails in the fluctuations distributions characteristic of non-equilibrium small systems (e.g. cells). As we have already discussed the effect of these fluctuations could be taken into account by considering their effect in the chemical potentials [3] which we must do as follows: The term relating mRNA *flows* due to transcriptional regulation could be written as a product of extended fluxes 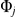 and forces 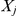. Here 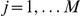 refers to the different mRNA species being regulated, that is, indexes 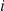 and 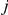 refer to the very same set of mRNA transcripts but in one case (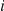) we take into account their local equilibrium behavior (as given by their independent chemical potentials and average local concentrations) and in the other case (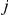) we are interested in their highly fluctuating (far from equilibrium) behavior as given by the term 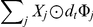.

#### Hyperbolic transport processes in non-equilibrium thermodynamics

It is known that the functions of genes that act as Transcription Factors (TFs) and genes that are expressed by the chemical action of such TFs (called Target Genes, TGs) are different. Research in the energetics of transcriptional regulation has suggested a noticeable difference between the chemical affinities between TFs and TGs [26], [27]; making that, in general, it is easier (less costly in energetic terms) to transcribe TFs than TGs. It has been discussed that TFs are genes whose expression is regulated by lower activation-energy barriers. TFs are involved in the transcriptional activation of other genes, then it is expected that they are synthesized first when energy is started to being released by metabolic processes within the cell. TGs should, in general be produced later and with higher activation energies thus leading to the role of TFs as master regulators of whole-genome expression. Now we need to propose a form for the extended fluxes and forces within this highly fluctuating regime, that at the same time allow for experimental verification, is simple enough to be solved and it is compatible with the axioms of extended irreversible thermodynamics. Here we are proposing a system of linear (in the forces) coupled fluxes with memory that was used to successfully characterize another highly fluctuating system, a fluid mixture near the critical point and gave rise to hyperbolic type (causal) transport equations [28]. The constitutive equations are,
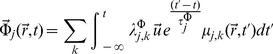
(13)

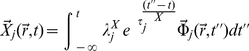
(14)


The 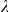
's are time-independent, but possibly space-dependent amplitudes, 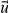 is a unit vector in the direction of mass flow (the nature of 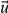 will not affect the rest of our description, since we will be dealing with the magnitude of the mass flux 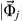) and 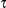
's are the associated relaxation times considered path-independent scalars. Since we have a linear relation between thermodynamic fluxes and forces some features of the Onsager-Casimir formalism will still hold. This will be especially important when considering cross-regulatory interactions. It can be shown that Equations 13 and 14 are mathematically equivalent to a system of hyperbolic differential (transport) equations (HDE) [29]. By definition, in an HDE, the Cauchy problem can be locally solved for any initial data along an arbitrary non-characteristic hyper-surface [30]. The solutions of HDEs are thus *waves*, i.e. when a disturbance is made in the initial data not every space-point registers the disturbance at once. Relative to a fixed time coordinate, disturbances have a *finite propagation speed*. This means that the non-singular solutions of *Cauchy Problems* in HDEs are causal. In fact, it is only natural to expect a lapse of time (lag) between synthesis of transcription factors and transcription mediated by these. This dynamic coupling is modeled by Eq. 13 and 14. Due to the spatial nature of the experimental measurements (either RNA blots or DNA/RNA chips and even present-day RNA-Seq techniques, measure space-averaged mRNA concentrations) it is possible to work with the related scalar quantities instead.
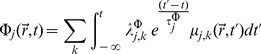
(15)

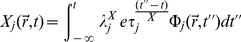
(16)


We could see that the energetics related to transcriptional regulation, as given by the third term in the r.h.s. of Eq. 12, namely 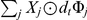 depends via equations 15 and 16 on the *transcription regulation chemical potentials* 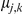 [2] as well as experimental parameters like the relaxation times (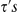 ) and the amplitudes (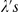). So whenever we know the dynamics of these energetic contributions 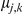 we are in a position to describe the dynamics of gene regulation as given by equation 12. By considering the effect of *fast processes* in a mesoscopic scale by means of generalized chemical potentials and their corresponding transport processes, we are taking an approach which is similar in philosophy to that of MNET [3], although of a less formal nature.

### Three simple mathematical models

In order to test for the applicability of the afore mentioned hypothesis we will introduce some *models* intended as working-examples. It is no-wonder that the dynamics of the regulatory chemical potentials could be quite complex, however we could probe the behavior of cell systems by considering simple models that however capture at least a part of the associated complexity. In the present work we will consider three different and, to greater extent complementary scenarios: an instantaneous pulse-like burst of energy, a cyclic energy release and an activated kinetic process. These are to be modeled as a delta function, a sinusoidal signal and a sigmoidal (hyperbolic tangent) process, respectively.

#### Instant pulse perturbation

Consider a Dirac distribution of the following form:
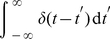
(17)


Delta distributed stimuli or transcriptional pulses are present in both, regular and anomalous, transcriptional bursts [31]–[37]. For delta functions we have the well-known result
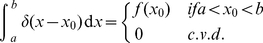
(18)


In view of this, the integrated effect of an instantaneous (delta-like) perturbation is:
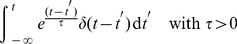
(19)


By changing variables to 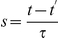, the integral in equation (19) becomes 
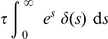
. So that we have:
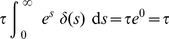
(20)


By introducing such result in equation 15, and by having in mind that in our case 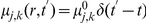, with 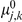 a constant, we obtain:
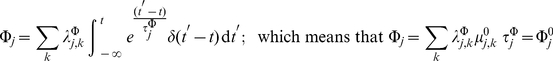
(21)and now, introducing this result into equation 16
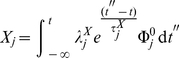
(22)


Now, let us consider the following change of variables 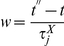, or 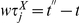; hence
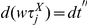



The integral in equation 22 becomes:
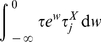
(23)


Substituting,
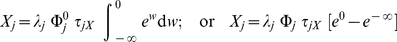
(24)to obtai
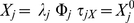
(25)


Hence, in the case that 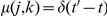 we get that
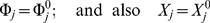
(26)


This means that the time-integrated effect of an instantaneous free energy pulse, reduces to the general case of a constant transcriptional flux and driving force [2]. This may seem a little bit counter-intuitive to molecular biologists, because in some instances they have assumed that pulses in energy influx (i.e. 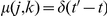) will necessarily imply pulses in transcriptional fluxes, something which do not happen due to memory effects (lags in the system's response). This is so, because by considering memory effects (i.e. a *kernel* such as the exponential one in equations 15 and 16) one is precluding the possibility of *parabolic transport processes* which are not of a causal nature and are thus un-physical [29].

#### Periodic biochemical stimuli

Now, let us turn our attention to a periodic chemical potential 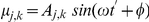 a case related to cyclic metabolic processes [38]–[44] with 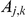 a system specific constant. In such case the integral in equation 15 is given by:
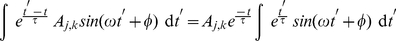
(27)


If we call 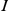 to the expression in the r.h.s. of equation 27, we can show that:
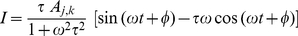
(28)


Let us see how; by substitution in Eq. 15 we get:
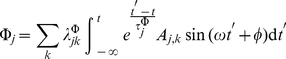
(29)

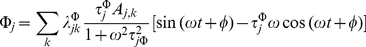
(30)


Now, if we insert equation 30 into equation 16 we obtain:
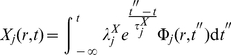
(31)

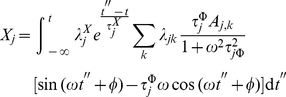
(32)

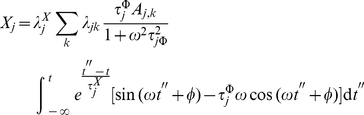
(33)

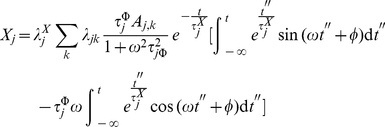
(34)


That is to say:
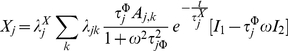
(35)where
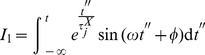
(36)and
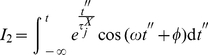
(37)


Solving 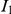 by partial integration we obtain
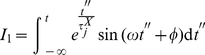
(38)

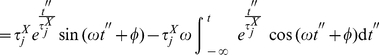
(39)


But this last integral equals 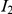 so we solve it by partial integration too
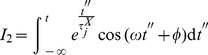
(40)

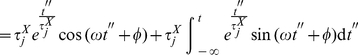
(41)


Then, we have
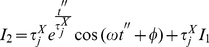
(42)


Hence
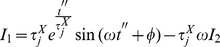
(43)

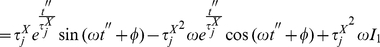
(44)


That gives
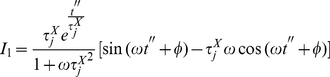
(45)


After solving both integrals by partial integration, their sum is given by:
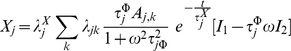


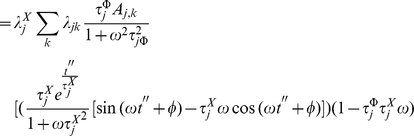


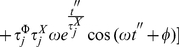
(46)


If we examine equations 30 and 46, we notice that periodic biochemical stimuli induces periodic transcriptional responses. These responses are (in spite of the nonlinearities) still coherent to the stimulus. Such *nonlinearities* reflect, although in a highly idealized manner, the effect of the lag in the response to transcriptional activation. In the results section we will discuss these phenomena more deeply.

#### Saturation kinetics

Let us now turn our attention to a model for saturation stimuli, namely that 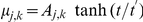. Hyperbolic tangent models in a simplified yet appropriate way those processes which start to grow gradually due to activation kinetics, then enter into a regime of constant grow and finally reach an asymptotic behavior due to saturation. By substituting this hyperbolic tangent model in equation 15 we obtain:
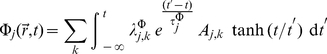
(47)


Since there is no closed, analytical solution of the integral in equation 47, we will resort to numerical estimates to it. Due to the singular behavior of the integrand, we introduced a cut-off time in the lower limit (instead of setting it equal to 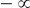), based on asymptotical considerations. Numerical integration was performed by means of the [R] mathematical and statistical computing system, using an implementation of the QUADPACK algorithm by Piessens, et al [45] that integrates by performing an adaptive quadrature of functions of one variable over a finite or infinite interval with extrapolation by the Epsilon algorithm and globally adaptive interval subdivision. More details in the results section below.

## Results

### Model results

It is known that the process of transcribing a gene can be divided roughly into three phases: initiation, elongation and termination. The initiation step involves the organization of the transcriptional complex onto the duplex genome at the promoter site for the specifically activated gene. The elongation step involves rate constants 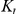 between 30 and 100 Hz and occurs at high ribonucleotide triphosphate concentrations (i.e. in a high free energy supply environment due to intense metabolic activity ). In the case that a Pyrophosporolysis reaction is involved the rate constants are also between 30 and 100 Hz, whereas the termination step is usually characterized by more moderated rate constants in the 0.1–1 Hz range. A *typical* gene (if such a thing it exists) would take on average up to some 2 minutes to be transcribed, hence 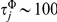 seconds [46]. Transcriptional processes are assumed as first order kinetic processes, hence within a single step sscenario, they may be described by Arrhenius type relations 
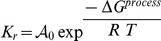
. If we consider a conservative value for the pre-exponential factor 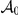 of, say 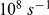 then, 
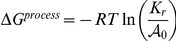
 would give us 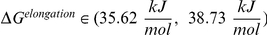 and 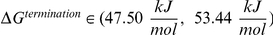. With regards to the initiation process, its energetics are more specific and system-dependent. Here we will consider an extremely simplified model with an approximate value of 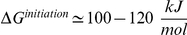. In such a manner that just for illustrative purposes the amplitude constant for transcription 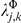 would be given by 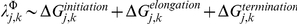. Given the figures just sketched, we have 
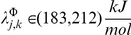
. So, for now on we will be taken 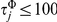 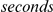, and 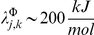.

#### Instant pulse

As we have already discussed, under a memory function formalism, the response of the gene-regulatory system to an instantaneous free energy supply is not that of a transcriptional pulse. A (small) constant transcriptional flux is established instead. In the highly simplified theory presented here, we are not yet taking into account degradation mechanisms that would modify the dynamics of mRNA level profiles. The reason is that nucleic acid degradation is controlled usually by means of *hunter* proteins which act upon post-translational modifications difficult to model. First of all, because the actual mechanisms of activation of these *degraders* are still under scrutiny [47] and second because experimental information about the dynamics of RNA degradation are not still available.

#### Periodic stimuli

**Figure 1:**
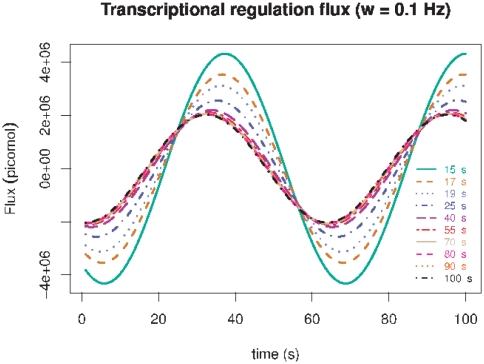
Transcriptional regulation flux (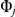) dynamics for periodic biochemical stimuli with 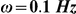. Different lines indicate different values of the associated relaxation time 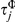 in seconds.

**Figure 2:**
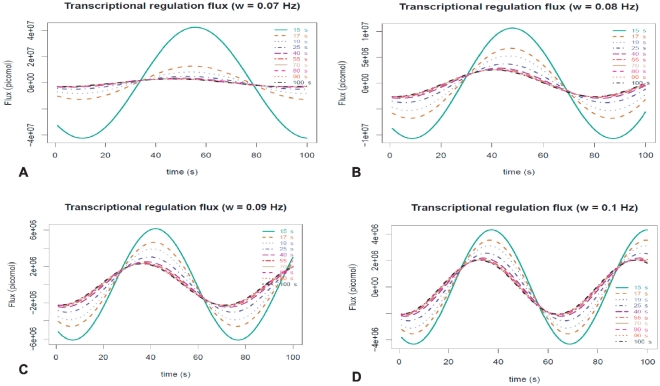
Transcriptional regulation flux dynamics for periodic biochemical stimuli for several values of frequency 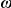. Panel A 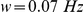, Panel B 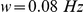, Panel C 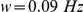, Panel D 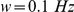 (same as previous figure). Different lines indicate different values of the associated relaxation time 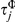 in seconds.

In Figure 1 we show the time evolution of the transcriptional flux 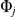 (i.e. the amount of mRNA for gene 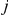 released per unit time) under the periodic stimuli model as given by equation 30. The model almost preserves the periodicity of the original energy influx (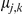) yet with a slight delay (or lag) dependence on the relaxation time of transcription (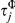), as it could be seen in the phase shift that different relaxation times curves (from 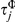 = 15 to 100 seconds) show in Figure 1. It is also possible to notice from Figure 1 that smaller transcriptional relaxation times induce higher transcriptional fluxes (as it is expected since genes regulated faster, could be transcribed more often in a given time lapse). In Figure 2 we could notice that the aforementioned effect of the relaxation time on the flux is now supplemented with an additional frequency (of the energy uptake) dependence. This effect is even more dramatic than that of the relaxation times, since a relatively small variation in frequency from 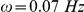 (panel A) to 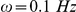 (panel D) induces a tenfold change in the maximum transcriptional flux (It should be noted that negative values of the flux are *un-physical* because when there is no free energy intake, transcriptional regulation -which is an activated process- simply do not proceed. Thus, only positive values of 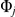 are to be considered on a physicochemical discussion.). Faster metabolic energy release dynamics give rise to smaller transcription fluxes due to a dynamic coupling effect which is modulated by the relaxation times 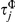. In other words, even if the metabolism is pumping energy faster to the system, the rate of transcription is limited by transcription factor dynamics and not only by energy availability. It is interesting to see that even in such a simplified model for the coupling between metabolism (as given by the release of energy that plays the role of a source of free energy, 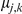) and transcriptional regulation (given by transcriptional flux 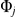 i.e. the rate of release of a given transcript 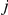 regulated by transcription factors 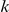) the fundamental features of the phenomena show up, namely the interplay between energy and time that are crucial to determine both the biochemical kinetics and its associated dynamics. The key factors in this model are thus the free energies and the relaxation times.

**Figure 3:**
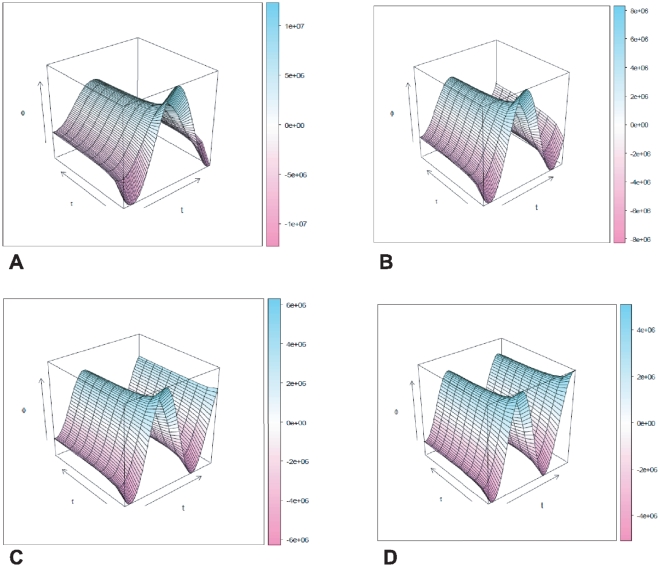
3D Plot of the relaxation-time dependent transcriptional regulation flux dynamics for periodic biochemical stimuli for several values of 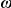. Panel A 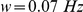, Panel B 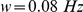, Panel C 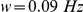, Panel D 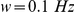.

**Figure 4:**
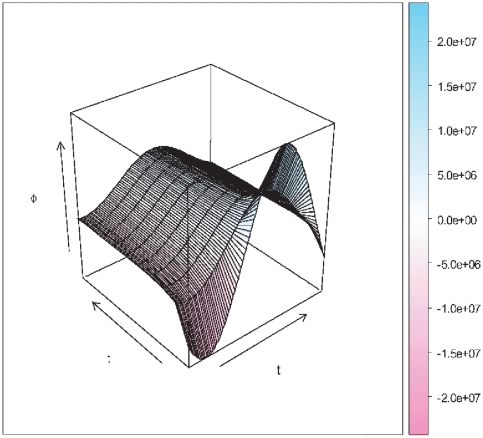
3D Plot of the relaxation-time dependent transcriptional regulation flux dynamics for periodic biochemical stimuli for 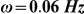.

**Figure 5:**
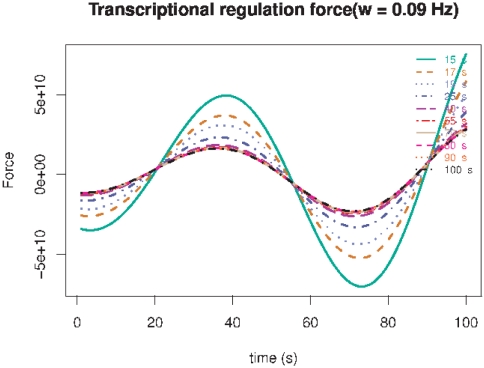
Thermodynamic flux-conjugate (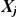) force dynamics for periodic biochemical stimuli with 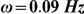. Different lines indicate different values of the associated relaxation time 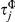 in seconds.

In order to appreciate better the nature of such dynamic coupling effects in the transcriptional flux, Figure 3 shows a 3D plot with both the relaxation-time and time dependence of 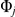 for different values of the frequency of energy release 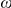. There we appreciate that the biggest effect is due to the frequency and also that the effect of the relaxation times is diminished at larger frequencies. However, if we look at the very-small relaxation times region of the plot (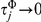), the effect of the relaxation times in *increasing* the flux is stronger. In Figure 4 we will see a closer look at this effect. The effect of short-relaxation time enhancement of the transcriptional flux is more dramatic in the low frequency domains, so we are plotting the behavior for 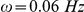. In Figure 5 we can observe that the behavior of the flux-conjugate (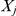) force dynamics is pretty similar to that of the flux, an expected result given the linear character of equations 15 and 16.

#### Saturation kinetics

**Figure 6:**
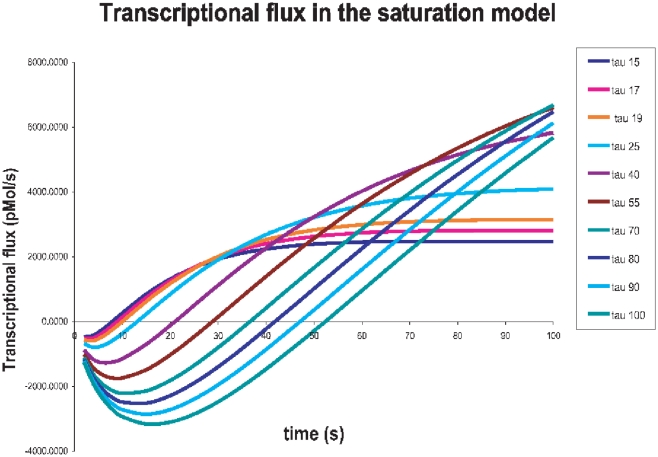
Transcriptional regulation flux dynamics for saturation kinetics. Different lines indicate different values of the associated relaxation time 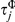 in seconds.

**Figure 7:**
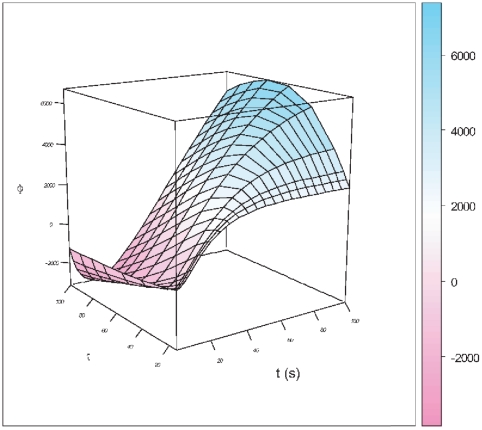
3D Plot of the relaxation-time dependent transcriptional regulation flux dynamics for saturation kinetics.

Numerical solutions of equation 47 have been obtained for different transcriptional relaxation times from 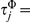
15 to 100 seconds. Results are shown in Figure 6. We could notice that for fast processes (i.e. processes with comparatively small values of 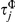, say 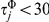 seconds) the activation phase occurs very quickly (as it is obvious), nevertheless saturation is also attained in a shorter time and maximum transcriptional fluxes are smaller than in the case of larger transcription relaxation times (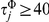 seconds). For these latter case we have found that the activation process take more time, but once activation is attained there is a longer lasting growing stage and in some instances saturation didn't even occurred within the considered time range. Maybe we could have a clearer picture by considering Figure 7, which is a 3D plot of the same results. As in the periodic signal we should take into account that only positive values of the transcriptional flux 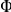 are *physical*, then the behavior observed would be as follows: in the case of shorter relaxation times one could notice a steeper activation stage followed by a fast growing stage that soon reaches saturation (steady state fluxes); whereas in the case of larger relaxation times what one could observe is a latency time with no flux, followed by a moderate growing stage, that is however, longer lasting than that for smaller relaxation times and it is prolonged so much that we could not see saturation reached during the time range under consideration.

## Discussion

### Experimental evidence for Anomalous Transcriptional Bursts

Experimental techniques in genomics are rapidly evolving, in such a way that probing the cell in real time under almost *in vivo* conditions is now becoming possible. In particular with regards to experimental verification of our models, there have been several instances in which related work has been done. One approach to provide real-time semi-quantitative analysis of transcription is the imaging of reporter gene expression, for example, using firefly luciferase [48], [49], such studies bring evidence to the hypothesis that gene expression is very dynamic over large periods and occurs in transcriptional bursts of varying duration that are not coordinated between different cells [50]. However, understanding real-time dynamics by direct quantification of transcription rates of multiple genes over time in single cells has not been achieved yet.

As we have already mentioned, the energy stimulation mechanisms just proposed are just highly idealized approximations to real phenomena. However, it still has been possible to observe experimental situations in which these approximations seem to hold, or at least seem to represent qualitatively the system's behavior. Transcriptional bursting activated by pulse-like stimuli have been observed in glandular expression [31] where pulses of light induce the expression of hormone response related genes in rats. *Chemical pulses* in which sudden release of a chemical stimulant produces the expression of the Gonadotrophin gene (another important hormone in mammal metabolism and signalling pathways) is reported in [32]. Even sets or networks of genes have been found to present transcriptional response to instantaneous perturbations [34]–[36]. It is noticeable that the examples from the literature correspond to genes associated to either signalling or hormone-related pathways.

In the case of periodic or quasi-periodic stimulation we have found that it has been reported in the context of cell synchronization even in species so *simple* as algae [38] and protozoa [39] but also in higher species like yeast [43], [44] and even mammals [42]. Different techniques have been used [39]–[41]. The very existence of gene clocks and circadian rhythms point-out to the presence of these oscillatory expression patterns (see reference [27] and references 8,9 and 14 therein) that are thus extremely important to understand time-regulated biological processes.

Saturation kinetics are of course extremely common in biochemical processes, from enzyme kinetics to protein complex formation to morphogenesis. In particular, sigmoidal saturation kinetics in gene expression have been observed in such disparate scenarios as cis regulation in chordates [51] (in this case in the highly conserved HOX gene family, extremely important in embryonic development, cell differentiation and morphogenesis), transport enhanced expression in rat retina [52] and thyroid hormone -mediated expression in glial cells [53], and are thus expected processes in many other instances [54].

### General discussion, importance, principal findings and perspectives

It is of course of general interest in current genomic studies to relate temporal patterns of gene expression associated with, for example, different developmental stages or disease conditions to study patterns of long-term developmental gene regulation either in homeostatic or in pathological conditions [55], [56]. Gene expression dynamics is central also to understand transcriptional regulation, it has been stated [57] that in order to express specific genes at the right time, the transcription of genes is regulated by the presence and absence of transcription factor molecules, but because of transcription factor concentrations undergoing constant changes, gene transcription takes place out of equilibrium undergoing complex dynamics [58], [59]. These changes may be related with signaling processes [60], promoter architecture [61], and many other biological processes. However, some of the most important changes in expression dynamics are due to the interaction between metabolism and transcriptional regulation [62] now commonly linked to extreme transcriptional de-regulation and cancer [63], [64]. It is no wonder that gene expression and metabolism have strongly tied connections but the explicit physicochemical mechanisms remain largely unexplored. Some studies have focused in network based semi-quantitative models of *cross-talk* interactions [65]; other studies aim to determine the extent to which the different levels of metabolic and transcriptional regulatory constraints determine metabolic behavior by means of a new flux-balance analysis under steady state conditions [66], in some instances, simultaneous measurements of cyclic AMP and gene expression for selected genes revealed a suspected relationship between specific gene expression and metabolism [67], [68].

One paradigmatic example of the tight relationship between changes in metabolism and gene expression levels is that of tumor cells. It is known that tumors could depend on energy production pathways that are different from those of normal cells. These unique pathways require in some cases the expression and function of so-called *tumor-specific enzymes*. Some of these glycolytic enzymes, as well as other modulators of tumor behavior, have recently been analyzed in search for a clue that inhibition of such enzymes or appropriate tuning of such modulators should deprive tumors of energy, while leaving nontransformed cells unaffected. Recent findings seem to point out to several so-called *metabolic transformations* that permit neoplasms survival, thus suggesting a role of metabolic pathways as potential pharmacological targets [69]. In fact, preliminary experiments on animals with hepatocellular carcinoma have indeed shown very encouraging results. It appears that modulating the energy production pathways of tumors is poised to become a substantial research area for cancer treatment [70].

In view of these hallmarks it is thus of foremost importance to have quantitative models, firmly founded in a physicochemical description, to probe for the behavior (although in a highly simplified manner yet) of genomic systems. Within this setting, we have found (for three different general models) the dependence that the amount of mRNA transcribed per unit time (what we call the transcriptional flux) have in the associated relaxation times and other kinetic parameters (activation energy amplitudes, frequency of periodic energy oscillations and so on) under a mandatory assumption of causality, i.e. as modeled by means of hyperbolic equations or memory functions. We are confident that such simplified description could serve as a basis for more detailed systematic studies that will help to unveil the role of thermodynamic processes in transcriptional regulation, and to ultimately understand the relationship between energetics and cell functioning.
